# An Ultrasonication-Assisted Green Process for Simultaneous Production of a Bioactive Compound-Rich Extract and a Multifunctional Fibrous Ingredient from Spent Coffee Grounds

**DOI:** 10.3390/molecules30153117

**Published:** 2025-07-25

**Authors:** Jaquellyne B. M. D. Silva, Mayara T. P. Paiva, Henrique F. Fuzinato, Nathalia Silvestre, Marta T. Benassi, Suzana Mali

**Affiliations:** 1 Department of Food Science and Technology, CCA, State University of Londrina, Londrina 86057-970, PR, Brazil; jaquellyne.bitten@uel.br (J.B.M.D.S.); martatb@uel.br (M.T.B.); 2 Department of Biochemistry and Biotechnology, CCE, State University of Londrina, Londrina 86057-970, PR, Brazil; mayara.thamela@uel.br (M.T.P.P.); henrique.fuzinato@uel.br (H.F.F.); nathalia.silvestre@uel.br (N.S.)

**Keywords:** agroindustrial residue, caffeine, melanoidins, antioxidant capacity

## Abstract

Spent coffee grounds (SCGs) are lignocellulosic residues generated from producing espresso or soluble coffee and have no commercial value. This study aimed to develop a new single-step process for extracting bioactive compounds from SCGs based on ultrasonication in an aqueous medium and simultaneously recovering the residual solid fraction, resulting in the integral utilization of the residue. This process resulted in a liquid aqueous extract (LAE) rich in bioactive compounds (caffeine: 400.1 mg/100 g; polyphenols: 800.4 mg GAE/100 g; melanoidins: 2100.2 mg/100 g) and, simultaneously, a solid multifunctional ingredient from modified spent coffee grounds (MSCGs) rich in bioactive compounds and dietary fibers (73.0 g/100 g). The liquid extract can be used as a natural ingredient for drinks or to isolate caffeine, while the solid matrix can be used to produce functional foods. This technique proved to be a promising eco-friendly alternative for the simultaneous production of two different materials from SCGs, maximizing resource efficiency, with some advantages, including short time, simplicity, and cost-effectiveness; using water as a solvent; and requiring no further purification processing.

## 1. Introduction

Coffee is a commodity of great economic importance worldwide and in Brazil, which is the world’s largest producer and exporter of green coffee beans. Coffee consumption is crucial in the global and Brazilian economies. The main types of beans produced are arabica (*Coffea arabica* L.) and robusta (*Coffea canephora* Pierre) [1,2,3,4,5].

According to the International Coffee Organization, global coffee consumption exceeded 177 million 60 kg bags in 2023/24 [2]. It is estimated that between 1.4 and 2.0 billion cups of coffee are consumed daily worldwide, with approximately 50 million produced using the espresso method [1,2].

Espresso coffee is quickly extracted using automatic machines, which produces a beverage characterized by its high soluble solid content [3,6,7]. From this solid–liquid extraction, large quantities of residue known as spent coffee grounds (SCGs) are obtained, and it is estimated that large coffee shops produce around 750,000 kg of this residue daily [8]. It was reported that 30–35% of the mass of coffee beans can be extracted during espresso preparation, and the remaining fraction ends up as SCGs, a residue with no commercial value [7,9,10,11].

SCGs can be defined as lignocellulosic residues with higher contents of bioactive compounds, including caffeine and polyphenols, which are well known for their antioxidant activity. SCGs consist primarily of total dietary fibers (43–64%), mainly insoluble dietary fibers (between 35–57%), including hemicellulose (20–39%), lignin (19–29%), and cellulose (12–24%), as well as soluble dietary fibers (~2–9%). It also contains approximately 4–11% protein, 2–15% lipids, and 0.7–4% minerals [6,12,13,14,15,16,17,18,19,20,21,22].

SCGs are rich in bioactive compounds with antioxidant activity and dietary fibers, and the valorization/transformation of this residue is aligned with the biorefinery and circular bioeconomy concepts by proposing the use of integrated processes to obtain different potential biobased products from a single raw material. Additionally, transforming SCGs meets the goals of the United Nations Sustainable Development Goals [20,21,22,23]. However, its use remains a challenge and requires physical, chemical, or biological treatments that can transform it for use in various industrial sectors, including the food sector. Conventional extraction processes, which utilize organic solvents, such as ethanol, methanol, and acetone have been frequently reported in the literature [9,14,21,22,23,24]. According to Arias et al. [22], using water as a solvent can be considered a feasible strategy to obtain extracts rich in bioactive compounds from SCGs with lower costs and minimal environmental impact.

Additionally, combining environmentally safe techniques, such as ultrasonication, can enhance the efficiency of extracting bioactive compounds. Ultrasonication has been reported as a simple technique that can use aqueous solutions for the extraction of bioactive compounds, being safe, fast, versatile, and applicable to different lignocellulosic matrices, allowing researchers to obtain high yields with the low generation of effluents [9,14,21,22,23,24,25,26,27,28,29,30,31].

Ultrasonic energy can be attributed to thermal and acoustic effects related to the proliferation of shock waves created by the cavitation effect [27,29,30,31]. This energetic effect also causes physical and chemical changes in lignocellulosic matrices that can affect their nutritional and techno-functional properties [28,31].

There are few authors reporting the modification or extraction of bioactive compounds from SCGs by ultrasonication in an aqueous medium [30,31]; despite this, both processes, modification or extraction, are carried out separately. The worldwide availability and economic importance of coffee make this residue an attractive raw material for exploitation through various approaches. This study aimed to develop a new single-step process for extracting bioactive compounds from SCGs based on ultrasonication in an aqueous medium while simultaneously recovering the residual solid fraction to obtain a multifunctional food ingredient, resulting in the integral utilization of the residue. This process is original and unique in the literature and resulted in two products: a liquid aqueous extract (LAE) rich in bioactive compounds with antioxidant capacity, and a solid multifunctional ingredient from modified spent coffee grounds (MSCGs) rich in bioactive compounds and dietary fibers.

## 2. Results and Discussion

### 2.1. Bioactive Compounds and Antioxidant Activity of Spent Coffee Grounds (SCGs), Liquid Aqueous Extract (LAE), and Modified Spent Coffee Grounds (MSCGs)

Table 1 presents the results of the quantification of bioactive compounds and the antioxidant activity of SCGs, the LAE, and MSCGs. Bioactive compound quantification was performed in the extract samples. (1) In SCGs, the bioactive compounds were extracted using a conventional approach with water (80 °C/10 min) as a solvent [32]. (2) In the LAE, the active compounds were quantified in the liquid extract sample collected by centrifugation after SCGs have been subjected to ultrasonication in an aqueous medium. (3) In MSCGs, the solid fraction recovered by centrifugation after the SCG sample had been subjected to ultrasonication in an aqueous medium, the bioactive compounds were extracted using water (80 °C/10 min) as a solvent [32].

As observed in Table 1, raw SCGs (100% arabica) presented 519 mg/100 g of caffeine (CA), 1380 mg GAE/100 g of total polyphenol content (TPC), and 6640.8 mg/100 g of melanoidins (MEs). The CA, TPC, and ME contents observed for SCGs in this study were similar to those values reported by other authors [33,34,35,36,37,38,39,40,41,42]. The composition of SCGs can vary significantly depending on the coffee variety and various external factors, including the cultivation conditions, geographic location, and climate conditions, among others [21,22,23,24]. The extraction efficiency strongly depends on the type of solvent, the extraction time, and the temperature [24,43].

Some authors reported higher values for CA in SCGs, such as Chatzimitakos et al. [33], who reported values of 660 mg/100 g for SCGs (60% arabica and 40% robusta), while Vandeponseele et al. [42] reported a large variation in CA contents (280 to 400 mg/100 g; 80% arabica and 20% robusta) depending on extraction parameters. The CA concentration depends on the type of bean, the origin, and the extraction method [4]; the levels of CA and TPC are reported to be higher in robusta coffee compared with arabica [4,23,25,44]. CA levels vary from 800 to 1400 mg/100 g and from 1700 to 4000 mg/100 g for arabica and robusta green coffee beans, respectively [25].

It is important to highlight that both the LAE and MSCGs had similar CA contents (Table 1), and that the sum of their contents was higher than the CA content observed in the raw SCG sample. This effect can be attributed to the ultrasonication process, which alters the structure, porosity, and swelling capacity of the lignocellulosic matrix, thereby facilitating the permeation and diffusion of solutes in the solvent. The lignocellulosic matrix of SCGs tends to collapse under ultrasonic waves resulting from cavitation effects, releasing bioactive compounds through mass transfer to the solvent by diffusion or dissolution, thereby maximizing extraction yields [27,28,29,30,31]. Okur et al. [29] reported that ultrasonication results in morphological changes on the solid matrix’s surface (micro-fissures), increasing the transfer of bioactive compounds into the solvent. According to Vandeponseele et al. [42], the efficiency of the extraction depends on the permeation of the solvent through the lignocellulosic matrix, followed by the solubilization of the solute (CA) and the diffusion of the solute through the solvent solution.

The CA content was 400.1 mg/100 g in the LAE (Table 1); this result was higher when compared with other authors that employed a conventional approach to obtain the extracts; Panusa et al. [44] carried out the extraction of bioactive compounds from SCGs (100% arabica) with 60% hydroethanolic solvent and pure water at a temperature of 60 °C for 30 min, obtaining a CA content of 96 mg/100 g and 97 mg/100 g, respectively. Vandeponseele et al. [42] reported CA values of 432 mg/100 g for extracts obtained from SCGs (80% arabica and 20% robusta) using a 60% hydroethanolic solvent, and 363 and 26 mg/100 g when employing water or ethanol as solvents, respectively, at a temperature of 20 °C for 15 min. Chatzimitakos et al. [33] reported values ranging from 170 to 568 mg/100 g for SCGs (60% arabica and 40% robusta), and higher values were obtained using water as a solvent for 120 min at 20 °C.

CA is a natural alkaloid that acts as a stimulating agent; it can be found in a wide variety of food products, including coffee, chocolate, teas, carbonated soft drinks, energy drinks, chocolate drinks, and fruit drinks. Traditionally, it has been used in painkillers and cosmetics [25,42]. In the last few years, the increase in caffeine consumption has led to a growing interest in the production of synthetic caffeine on an industrial scale, which is labor-intensive and results in high costs [33]. Considering the maximum recommended caffeine intake for an adult (400 mg/day) [25], both the LAE and MSCGs can be considered potential renewable sources of caffeine, especially for food-grade products, since no harmful reagents were employed in the extraction process.

As observed in Table 1, TPC contents were 1380.0, 800.4, and 821.0 mg GAE/100 g for the SCGs, LAE, and MSCGs, respectively. A large variation in TPC contents was observed in the literature for SCG samples. Chatzimitakos et al. [33] reported values of 1980 mg GAE/100 g for SCGs (60% arabica and 40% robusta), Seo and Park [34] also reported values of 1652 mg GAE/100 g for SCGs, Martínez-Inda et al. [36] reported values ranging from 336 to 1352 mg GAE/100 g (arabica and robusta mix), Papageorgiou et al. [37] reported values of 2160 mg GAE/100 g for SCGs (100% arabica), and Panusa et al. [44] reported polyphenol contents of 1745 mg GAE/100 g for SCGs obtained from 100% arabica coffee and 3552 mg GAE/100 g for SCGs from 40% arabica and 60% robusta coffee.

TPC contents for the LAE and MSCG samples followed the same trend observed for CA (Table 1). The sum of their contents was higher than the TPC content observed in the raw SCGs, which was attributed to the cavitation effect from ultrasonication. According to Solomakou et al. [1], an ultrasound-assisted process can accelerate solvent extraction. Fragmentation and erosion of the solid lignocellulosic matrix can enhance mass transfer, solvent penetration, and the release of intracellular components, resulting in improved extraction rates and yields, and reducing the need for organic solvents [23,29].

A large variation in TPC contents can be observed in the literature for SCG extracts, which is possibly attributed to the different extraction protocols and starting materials. Panusa et al. [44] employed water as a solvent in a conventional approach (60 °C; 60 min), and they reported lower values than those obtained in this study, with TPC values of 743 mg GAE/100 g from SCGs (100% arabica), while the TPC content was higher (1258 mg GAE/100 g) when using a 60% hydroethanolic solvent under the same conditions.

Chatzimitakos et al. [33] reported TPC values of 1985 mg GAE/100 g for SCGs (60% arabica and 40% robusta) using a 50% hydroethanolic solvent for 120 min at 65 °C. Sant’Anna et al. [45] extracted bioactive compounds from SCGs (100% arabica) with a boiling aqueous solvent (10 min); they obtained lower results than those described in our study, with 566 mg GAE/100 g.

Most published studies have reported the use of ultrasonication-assisted processes to obtain SCG extracts using ethanol, hydroethanol, methanol, or hydromethanol as solvents. Solomakou et al. [1] reported TPC values ranging from 285 to 1854 mg GAE/100 g for SCG (100% arabica) extracts obtained by ultrasonication (20 a 60 °C), and the higher values were obtained employing a 60% hydroethanolic solvent, which was reported by the authors as a more efficient strategy when compared with mono-component solvent systems. Al-Dhabi et al. [27] observed values ranging from 3231 to 3623 mg GAE/100 g for SCG extracts obtained by ultrasonication using ethanol as solvent (30–50 °C; 5–45 min). Okur et al. [29] reported values of 951 mg GAE/100 g for SCG extracts obtained by ultrasonication using methanol as solvent (25 °C; 15 min). Severini et al. [46] reported the extraction of bioactive compounds by ultrasonication using hydromethanol solutions as the solvent, and they observed TPC values ranging from 1929 to 2495 mg GAE/100 g in SCGs.

Zhang et al. [30] employed ultrasonication (55–75 °C; 25 min) and water as a solvent to obtain SCG extracts, and they obtained a similar TPC result (820 mg GAE/100 g) to that obtained in this study. Samsalee and Sothornvit [31] employed ultrasonication and water as a solvent, and they reported that processes carried out with 60% amplitude for 30 min produced significantly higher TPC levels compared with the conventional process.

Polyphenolic compounds are an important group of molecules with antioxidant capacity that are found abundantly in SCGs. Additionally, these compounds are also associated with several health-promoting effects, including anti-inflammatory, anticancer, antiglycating, and antihyperglycemic effects. Several industrial sectors can benefit from the use of these compounds, including food, medicine, and cosmetics, among others [21,22,23,24].

This study obtained a ME content of 6640.8 mg/100 g for SCGs (Table 1); after ultrasonication in an aqueous solvent, a reduction was observed in the ME contents of the LAE (2100.2 mg/100 g) and MSCG (3439.6 mg/100 g) samples (Table 1). Martínez-Inda et al. [36] reported the extraction of MEs by a solid–liquid process using hydroethanolic solutions (40 °C; 24 h, 250 rpm) as extracting solvents, with ME values ranging from 2225 to 24,435 mg/100 g for SCG extracts (arabica and robusta mix); the lower value was obtained when pure water was employed as a solvent, while the higher value was when a 75% hydroethanolic solution was employed.

MEs are high-molecular-weight nitrogen compounds produced at the end of the Maillard reaction process. They are associated with health benefits, including antioxidant, anti-inflammatory, antihypertensive, and antiglycation properties [47]. Additionally, they possess a high antimicrobial potential that can be utilized to inhibit the growth of pathogenic bacteria in food products [48]. Coffee is the largest source of MEs in the human diet [47]. Viencz et al. [43] reported levels between 10,800 and 17,600 mg ME/100 g in *Coffea canephora* from the Amazon region.

As observed in Table 1, SCGs presented 2015.2 and 267.4 mg TE/100 g of antioxidant activity detected by the DPPH and ABTS methods, respectively. These results were consistent with the literature. Xu et al. [39] obtained 1498 mg TE/100 g in SCGs, and Ballesteros et al. [40] reported values of 2815 mg TE/100 g. For the ABTS method, McNutt et al. [41] reported 396.2 mg TE/100 g, while Bijla et al. [15] reported values of 377.7 mg TE/100 g. The antioxidant activity of SCGs can be attributed to the presence of both polyphenolic compounds and melanoidins [36,38].

The antioxidant activity (DPPH and ABTS) significantly decreased for the LAE and MSCGs compared with the SCGs, and it was significantly higher for the LAE than for MSCGs (Table 1). Brzezińska et al. [49] obtained a result of 730 mg TE/100 g (ABTS) in a liquid extract obtained from SCGs subjected to an ultrasonication process in a 65% hydroethanolic solution. Sant’Anna et al. [45] used boiling water as a solvent and obtained 805 mg TE/100 g (ABTS). The higher results described in the literature are possibly related to the processing conditions (equipment, time, temperature, pressure) as well as the extraction solvent. On the other hand, Page et al. [50] produced an ethanolic extract from SCGs and obtained lower antioxidant activity (1.84–2.36 mg TE/100 g) (DPPH).

### 2.2. Chemical Characterization of the Solid Fractions: SCGs and MSCGs

The chemical compositions of the SCGs and MSCGs are presented in Table 2. The raw material (SCGs) was characterized mainly by its high content of insoluble fibers (64.1 g/100 g), including cellulose (14.1), hemicellulose (23.0), and lignin (36.0 g/100 g), and lipids (10.8 g/100 g). Coffee residues are rich in polysaccharides, with the major fraction consisting of insoluble fibers [51,52,53], and, according to Mussatto et al. [54], an important portion of the insoluble polysaccharides is retained in the SCG matrix after coffee beverage preparation.

Significant changes in chemical composition were observed (Table 1) when SCGs were subjected to ultrasonication in an aqueous medium to obtain the LAE and MSCGs. Moisture, ash, and lipid contents decreased, and protein, total, and insoluble fiber contents increased. The ash results found for SCGs (1.9 g/100 g) (Table 1) agreed with the values reported by Mata et al. [55], which observed ash contents between 0.4 and 2.2 g/100 g, and also by Girotto et al. [14], Mussatto et al. [54], Ballesteros et al. [40], Go et al. [53], which reported values ranging from 1.30 to 1.73 g/100 g of ashes in SCG samples. With the modification of SCGs, there was a 52.7% decrease in the ash content (0.92 g/100 g) (Table 2). The ultrasonication process, which breaks down the plant cell walls of the material and rearranges the fibrous structure, increases the contact surface between the solvent and the lignocellulosic matrix and, consequently, the leaching of soluble ashes [26,27].

Among coffee industry residues, SCGs can be considered the residue with the highest lipid content, ranging from 7.0 to 21.0 g/100 g, followed by silverskin (2.0–5.0 g/100 g), coffee hulls (1.0–3.0 g/100 g), and pulp (0.80 g/100 g) [56]. The result of this study (10.8 g/100 g) agreed with results obtained by other authors for SCGs, such as Mata et al. (10–15 g/100 g) [55], and it was lower than the values reported by Go et al. [53], which reported lipid contents of 13.8 to 16.5 g/100 g. SCGs present a higher lipid content compared with other residues, such as oat hulls (4.5 g/100 g) and soybean hulls (7.5 g/100 g) [57].

MSCGs had a 32.4% decrease in lipid content compared with SCGs (Table 2). This result is attributed to the ultrasonication process being effective in extracting lipids. It is possible that the ultrasonic waves released lipids by rupturing the cell membrane and altering its diffusion rate [27,42,58].

The protein content (1.2 g/100 g) (Table 2) obtained for SCGs was lower than the value reported by Valdés et al. [59], who observed a protein content of 2.9 g/100 g for SCGs obtained from espresso coffee; and it was also lower than values reported by other authors (4.5–17.3 g/100 g) [17,18,54].

MSCGs had a significantly higher protein content than SCGs, with a value of 2.9 g/100 g (Table 2), which can be attributed to the ultrasonication process at high power (20–100 kHz). Cavitation affects the surface area of cellular matrices in plant cells, altering the structural, physical, chemical, and functional properties of these materials by breaking hydrogen bonds and disrupting hydrophobic interactions [21,22,23,24,25,29,30,31].

The results for soluble fiber in this study (Table 2) were 1.4 and 1.6 g/100 g for SCGs and MSCGs, respectively. These results were lower than those observed by other authors, which ranged from 2 to 8 g/100 g [45,48].

According to the literature, the major component of SCGs is dietary fiber, particularly insoluble fibers (cellulose, hemicellulose, and lignin) [15,17,54]. SCGs showed a high content of total fibers (65.5 g/100 g) (Table 2), which agrees with Ballesteros et al. [40]. Benincá et al. [60] reported a lower content of 32%. When SCGs were subjected to ultrasonication in an aqueous medium, the total fiber content increased by 11.5% (73.0 g/100 g in MSCGs). Trà et al. [61] reported results similar to those found in this study (76.6 g/100 g). MSCGs presented a higher total fiber content than the other coffee residues reported by Oliveira et al. [62], such as silverskin (~62 g/100 g) and coffee hulls (~66 g/100 g).

The increase in fiber content in MSCGs can be attributed to ultrasonication, primarily caused by cavitation. Cavitation expands the structure of the fibers, allowing polar sites or entire molecules to interact with the solvent, resulting in changes in the morphological and chemical composition of these materials [21,22,23,24,25].

Insoluble fiber comprises the lignocellulosic complex (cellulose, hemicellulose, and lignin). After being subjected to ultrasonication, MSCGs differed statistically (*t*-test, *p* ≤ 0.05) from SCGs, resulting in an increase from 14.1 to 17.0 g/100 g for cellulose, from 23.0 to 27.4 g/100 g for hemicellulose, and from 36.0 to 39.1 g/100 g for lignin, an increase of 21.4%, 19.1%, and 8.6%, respectively (Table 2). According to the literature, the cellulose content of SCGs is between 12 and 24 g/100 g, hemicellulose is between 20 and 39 g/100 g, and lignin is between 19 and 29 g/100 g [6,12,15,17,18,54,55].

Insoluble fibers are essential for health because they can increase the fecal cake, improve intestinal peristalsis and evacuation, and have techno-functional properties in food, such as the ability to absorb water and oil, swelling, and enhancing the texture and stability of foods [57].

Studies have reported the potential of SCGs as a source of prebiotic compounds [36,48], mainly because of their insoluble fiber composition. Jiménez-Zamora et al. [48] and Panzella et al. [63] performed digestion and gastrointestinal fermentation in vitro and verified the high prebiotic activity of modified SCGs. This was attributed to the composition of the lignocellulosic complex, where the modification promoted the release of the lignin fraction, making it potentially bioaccessible after simulated digestion/fermentation. In addition, López-Barrera et al. [64] reported that the production of short-chain fatty acids by the partial fermentation of insoluble fibers in the gastrointestinal tract prevents colon inflammation [36]. Therefore, MSCGs can be used as a multifunctional ingredient.

Hemicellulose is a branched heteropolysaccharide composed of different monosaccharides depending on the botanical source, including glucose, mannose, galactose, xylose, arabinose, and glucuronic acid. In SCGs, hemicellulose is composed mainly of three sugars, namely mannose, followed by galactose and arabinose, forming galactomannans and arabinogalactans [41,42,45]. According to Mussatto et al. [54], the increase in SCGs after chemical or physical treatments is linked to the hydrolysis process, in which the components of the lignocellulosic complex are separated.

### 2.3. Techno-Functional Properties of the Solid Fractions: SCGs and MSCGs

The results of the techno-functional properties are shown in Table 3. No difference in OHC and HI was observed between SCGs and MSCGs, but the ultrasonic treatment statistically (*p* ≤ 0.05) increased WHC, SC, and EA.

The ability to hydrate, retain, and absorb solvents is physiologically and technologically relevant, especially for food applications [65]. WHC refers to liquids that are hydrodynamically bound to the fibers, even under the action of centrifugal forces, thus being influenced by the particle size, material porosity, the hydrophobicity of the compounds, and ambient temperature, which affects the successful incorporation of fibrous ingredients into foods [65,66]. WHC statistically increased (*t*-test, *p* ≤ 0.05) from 3.5 g/g in SCGs to 3.7 g/g in MSCGs (Table 2), which is consistent with the increase in fibers in the sample subjected to ultrasonication. Wang et al. [66] reported that dietary fibers are associated with nutritional value in food products, but they also affect functional and physicochemical properties.

Ballesteros et al. [40] and Silva et al. [57] reported that higher amounts of total dietary fiber result in materials with a higher WHC, agreeing with the results of this study. Vargas-Sánchez et al. [67] reported a higher WHC value for flake coffee silverskin (5.7 g/g) and a lower value for powder coffee silverskin (2.9 g/g), while Ballesteros et al. [40] reported a higher value for SCGs (5.73 g/g). Silva et al. [57] reported similar results for other residues (soybean hulls, coffee hulls, and oat hulls) treated with hydrothermal and chemical processes, obtaining materials with increased fiber contents and improved water absorption capacities.

The results of both the SCG (2.83 g/g) and MSCG (2.75 g/g) samples for OHC did not differ statistically (*t*-test, *p* ≤ 0.05, Table 3). These results were lower than those described by other authors, such as Ballesteros et al. [40] for SCGs (5.20 g/g) and coffee silverskin (4.72 g/g), and Vargas-Sánchez et al. [67] for flake coffee silverskin (5.29 g/g) and powder coffee silverskin (3.62 g/g).

The increase in SC from 3.9 mL/g of SCGs to 4.2 mL/g of MSCGs (Table 3) can be explained by the increase in the insoluble fraction of the fibers (Table 2), combined with the modification of the lignocellulosic matrix by ultrasonication [27]. The MSCGs’ results were comparable with those of coffee parchment (4.00 mL/g) [52]. Swelling is primarily a surface phenomenon, which increases as the interior of the structure becomes more hydrated, thus tending to swell and having an intimate relationship with water absorption capacity, being affected in the same way by the change in the structure of the fibers present in the matrix, where the polysaccharides can expose more water binding sites by aggregating the surrounding liquid molecules through hydrogen interactions. The fibers, especially the insoluble ones, form a hydrophilic matrix that allows water to be trapped, causing considerable swelling [52].

HI is a property that determines a material’s ability to adsorb moisture from the atmosphere, thereby influencing the potential for a product to exhibit greater or lesser storage stability under various environmental conditions [39]. The HI for SCGs and MSCGs was 11.0 and 10.8%, respectively (Table 2). The GEA Niro Research Laboratory [68] categorizes both samples as slightly hygroscopic powders; Silva et al. [57] reported that coffee hull is categorized as a hygroscopic powder with 20.5% hygroscopicity.

EA significantly increased (*p* ≤ 0.05) from 2.0% in SCGs to 3.3% in MSCGs (Table 3), and this possibly was related to the increase in the protein and hemicellulose contents observed in MSCGs compared with SCGs (Table 1). Proteins act by anchoring the moiety of fiber to the oil or water interface and arranging the hydrophobic and hydrophilic groups at the water–oil interface [31,40]. Olorunsola et al. [69] reported that hemicellulose is a complex polysaccharide with promising emulsifying properties superior to typical gums and that the EA of hemicellulose can be attributed to the viscosity modification of the dispersion medium.

### 2.4. Cholesterol (CAC) and Glucose (GAC) Adsorption Capacities of the Solid Fractions: SCGs and MSCGs

There was a significant increase (*t*-test, *p* ≤ 0.05) in CAC at pH 7 and GAC (50 mmol and 100 mmol) in MSCGs when compared with SCGs (Table 4). The increased ability to adsorb cholesterol and glucose was primarily attributed to the increase in fiber and protein content, as well as improvements in water holding capacity (WHC) and solubility capacity (SC) [66,67,68,69].

The CAC assay simulated the gastrointestinal environment in the human body. At pH 2, the simulation conditions mimicked those of the stomach, and the results in Table 3 show that SCGs adsorbed 2.7 mg of cholesterol/g, which was statistically higher (*t*-test, *p* ≤ 0.05) than MSCGs, which adsorbed 1.6 mg of cholesterol/g. However, at pH 7, where the conditions simulated cholesterol adsorption in the intestine, a 400% increase was obtained for MSCGs (8.0 mg cholesterol/g) compared with SCGs (1.6 mg cholesterol/g). Wang et al. [66] reported CAC values ranging from 1.7 to 10.9 mg/g at pH 2 and from 3.22 to 17.90 at pH 7 for citrus fruit fibers.

Glucose has a large potential to bind efficiently to samples with high insoluble fiber contents [52]. Table 4 shows that the results for GAC increased with the modification of SCGs for both glucose concentrations (50 mmol/L and 100 mmol/L). GAC (50 mmol/L) increased from 4.7 mmol/L in SCGs to 9.3 mmol/L in MSCGs, increasing by 97.9%; GAC (100 mmol/L) increased by 164.2% in MSCGs (Table 3).

Additionally, the increase in fiber content (cellulose and hemicellulose) also affected CAC and GAC. While soluble dietary fiber influences blood glucose regulation and cholesterol reduction by forming a gel and retaining water in the intestine, insoluble dietary fiber plays a crucial role in reducing cholesterol and blood pressure due to its swelling capacity and adsorption properties [66].

## 3. Materials and Methods

### 3.1. Materials

SCGs (100% arabica coffee with a light–medium roast degree) from espresso coffee were provided by a large coffee shop in Londrina, Paraná, Brazil. The residue was dried for 12 h at 45 °C in an air-circulating oven (Marconi MA 035, Marconi Company, São Paulo, Brazil) to a constant weight.

### 3.2. Ultrasonication-Assisted Process in an Aqueous Medium

**Figure 1:**
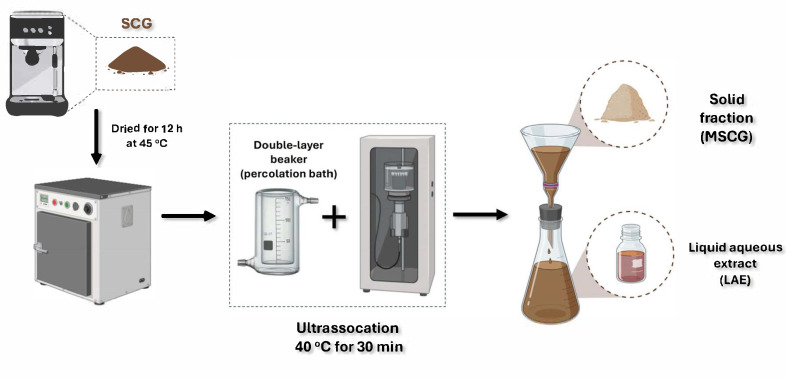
Flowchart of the ultrasonication process in an aqueous medium.

The modification of the SCGs was carried out according to Al-Dhabi et al. [27] and Okur et al. [29], with modifications, using an ultrasonicator (Model Q700 QSonica, QSonica, Newtown, CT, USA) with a frequency of 20 kHz, 700 W of power, and 60% amplitude, coupled with a probe with a tip diameter of 1.27 cm (Fisher Scientific Model FB 4219, Fisher Scientific, Pittsburgh, PA, USA). In a double-layer beaker, approximately 10 g of SCGs with 170 mL of water were subjected to ultrasonication, coupled to a percolation bath, at a constant temperature of 40 °C for 30 min. Subsequently, the suspension was vacuum-filtered, and the liquid aqueous extract (LAE) was collected to quantify bioactive compounds (caffeine, total polyphenols content, and melanoidins) and antioxidant activity based on radical scavenging capacity. The recovered solid fraction was dried in an air-circulating oven (Marconi MA 035, Marconi Company, São Paulo, Brazil) at 105 °C for 1 h to obtain the modified spent coffee grounds (MSCGs) (Figure 1).

### 3.3. Bioactive Compounds in the SCGs, LAE, and MSCGs

#### 3.3.1. Preparation of Extracts for Bioactive Compound Quantification

The liquid aqueous extract (LAE) after the ultrasonication-assisted process was used to determine caffeine, total polyphenol content, melanoidins, and antioxidant activity.

For the determination of caffeine, total polyphenol content, melanoidins, and antioxidant activity, SCG and MSCG samples were prepared according to Vignoli et al. [32] The raw SCGs (0.5 g) were mixed with 30 mL of distilled water at 80 °C for 10 min, followed by centrifugation (5804 R, Eppendorf, Hamburg, Germany) at 1000× *g* for 10 min. The supernatant was collected for analysis. For MSCGs, after the sample had been subjected to ultrasonication in an aqueous medium, this same protocol was employed.

#### 3.3.2. Caffeine (CA)

**Figure 2:**
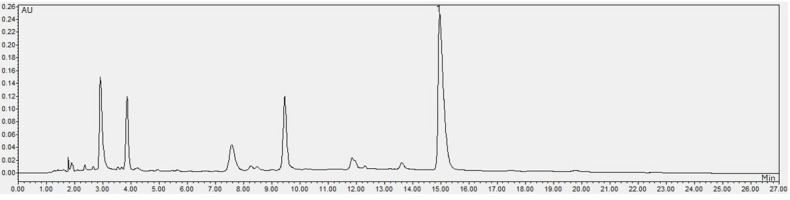
Chromatogram of caffeine in spent coffee grounds. (1) Caffeine peak at 272 nm.

**Figure 3:**
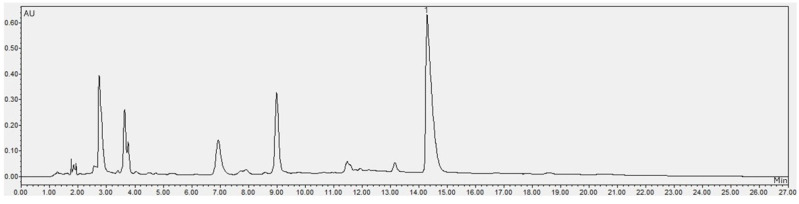
Chromatogram of caffeine in the aqueous liquid extract (used to quantify caffeine in the spent coffee grounds modified in an ultrasonicator and in the liquid extract). (1) Caffeine peak at 272 nm.

The caffeine content was determined using the methodology described by Portela et al. [70] in an ultra-high-performance liquid chromatograph (Waters Acquity, Waters, Milford, CT, USA). The samples were diluted in ultrapure water and filtered (0.45 µm nylon syringe). A Spherisorb ODS-1 column (150 × 3.2 i.d., 3 μm) (Waters, Milford, CT, USA), HPLC grade acetonitrile (Merck, Darmstadt, Germany), analytical grade acetic acid (Anidrol, Diadema, Brazil), and a caffeine standard (Sigma-Aldrich, St. Louis, MO, USA) were employed. The samples were eluted in a gradient elution of 5% acetic acid and acetonitrile, and the detection was carried out at 272 nm in a 27 min run. Quantification was performed using a 7-point standard curve (1 to 60 µg/mL). The result was expressed in mg/100 g of the sample. The chromatogram of CA in the raw SCGs and the chromatogram of CA in the aqueous liquid extract were used to quantify CA in the LAE and MSCGs and are presented in Figure 2 and Figure 3, respectively.

#### 3.3.3. Total Polyphenol Content (TPC)

TCP was determined using the Folin–Ciocalteu [71] spectrophotometric method using acid gallic as a reference. The absorbance was measured using a spectrophotometer (765 nm) (SL244 spectrophotometer, Elico, Hyderabad, India). TPC was expressed in mg of gallic acid equivalent (GAE) per 100 g of the sample.

#### 3.3.4. Melanoidins (MEs)

ME estimation was carried out according to Mori et al. [72] with modifications. Diluted extracts (4–10 mg/mL) were read in a spectrophotometer (SL244 spectrophotometer, Elico, Hyderabad, India) at a wavelength of 420 nm. ME content was estimated on the basis of the absorptivity value of 1.1289 L/g.cm. The results were expressed in mg/100 g of the sample.

#### 3.3.5. Antioxidant Properties: Free Radical Scavenging Activity

Determination of the free radical scavenging activity by the DPPH (2,2-diphenyl-1-picrylhydrazyl) method was carried out according to Blois [73] and Dinis et al. [74]. The reduction in the free radicals was determined by analysis in a spectrophotometer (SL244 spectrophotometer, Elico, India) at 517 nm. The antioxidant capacity of the samples was determined using a Trolox (6-hydroxy-2,5,7,8-tetramethychroman-2-carboxylic acid) analytical curve (2–5 mmol/L), and the result was expressed in mg Trolox equivalent/100 g of the sample (mg TE/100 g sample).

Determination of the antioxidant radical sequestering capacity by the ABTS (2,2′-azino-bis-3-ethylbenzothiazoline-6-sulfonic acid) method was carried out according to Re et al. [75] The calibration curve was made with a standard Trolox solution (1–8 mmol/L) and read in a spectrophotometer at 730 nm (SL244 spectrophotometer, Elico, India). The result was expressed in mg Trolox equivalent per 100 g of the sample (mg TE/100 g sample).

### 3.4. Chemical Characterization of the Solid Fractions: SCGs and MSCGs

#### 3.4.1. Chemical Composition

The chemical composition analyses (moisture, ash, lipids, proteins, total dietary fiber, insoluble dietary fiber, and soluble dietary fiber) were carried out according to the Association of Official Analytical Chemists (AOAC) [76].

#### 3.4.2. Cellulose, Hemicellulose, and Lignin

Cellulose and hemicellulose were quantified using the methodology described by Van Soest [77]. The insoluble lignin content was determined using the method of the Technical Association of the Pulp and Paper Industry—TAPPI test method [78].

### 3.5. Techno-Functional Properties of the Solid Fractions: SCGs and MSCGs

#### 3.5.1. Water Holding (WHC) and Oil Holding Capacity (OHC)

WHC and OHC were determined according to the methodology described by Benítez et al. [79], using distilled water (WHC) or soybean oil (OHC), and WHC was expressed in g H_2_O/g, and the OHC is expressed as g oil/g.

#### 3.5.2. Swelling Capacity (SC)

SC was determined on the basis of the methodology described by Mateos-Aparicio et al. [80], and SC (mL/g) was calculated from the volume (mL) occupied by the sample divided by the sample’s mass (g).

#### 3.5.3. Hygroscopicity (HI)

HI was quantified following the methodology described by Castro-Muñhoz et al. [81]. The percentage of hygroscopicity was calculated using Equation (1).

HI (%) = ((Wsf − Wsi)/Wsi) × 100(1)

where Wsi is the initial weight of the sample (g), and Wsf is the final weight of the sample.

#### 3.5.4. Emulsifying Activity (EA)

EA was determined according to Seibel et al. [82]. Each sample (1.0 g) was mixed with 10 mL of distilled water and 10 mL of soybean oil, agitated in a turrax (Turratec TE-102, Tecnal, Piracicaba, Brazil) for 1 min at 1000× *g*, and then centrifuged (5804 R, Eppendorf, Hamburg, Germany) (1000× *g*) for 5 min. The emulsification layer was measured, and EA was calculated using Equation (2).

EA (%) = (Vel/Vi) × 100(2)

where Vel is the emulsification layer of the sample (mL), and Vi is the initial volume of the suspension (mL).

### 3.6. Cholesterol (CAC) and Glucose (GAC) Adsorption Capacities of the Solid Fractions: SCGs and MSCGs

CAC was quantified according to the method described by Daou et al. [83], with some modifications. Fresh egg yolk was diluted (1:9) in distilled water, and then 1.0 g of each sample was weighed and added to the egg yolk dilution (±25 mL). The pH was adjusted to 2.0 and 7.0, and the samples were incubated at 37 °C for 2 h. After homogenization and centrifugation (5804 R, Eppendorf, Hamburg, Germany) at 3500× *g* for 10 min, CAC was determined using the GOD-PAP triglyceride enzyme kit (Laborlab S.A, Guarulhos, Brazil) for each pH condition. CAC was calculated according to Equation (3) and the result was expressed in mg/g

CAC (mg/g) = (TGi − TGaf)/M(3)

where TGi are the initial triglycerides present in the sample without incubation, TGaf is the triglycerides of the final absorption in the sample after incubation, and M is the mass of the sample.

GAC was determined according to the method described by Benítez et al. [52]. Each sample (1.0 g) was added to 100 mL of a glucose solution at different concentrations (50 and 100 mmol/L). The mixtures were then incubated at 37 °C for 6 h and then centrifuged (5804 R, Eppendorf, Hamburg, Germany) for 15 min at 3500× *g*. The supernatant was collected to quantify adsorbed glucose by the 3,5-dinitrosalicylic acid (DNS) method [84], and the result was expressed in mmol/L.

### 3.7. Statistical Analysis

The results are expressed as mean values ± standard deviation. For means comparison, *t*-test and Tukey’s test were employed with a significance level of 5% (*p* ≤ 0.05) using R software 4.5.0 (R Foundation for Statistical Computing, Vienna, Austria).

## 4. Conclusions

The modification of SCGs employing an ultrasound-assisted process in an aqueous medium proved to be promising for simultaneously obtaining (1) a liquid aqueous extract and (2) a solid lignocellulosic matrix rich in fibers, both rich in caffeine, polyphenols, and melanoidins, with antioxidant activity, promoting the full use of the residue. The liquid extract can be used as a natural ingredient for drinks or to isolate caffeine, while the solid matrix can be used to produce functional foods, supplements, or feed. Ultrasonication was able to improve the techno-functional properties of MSCGs, with positive effects on its nutritional properties, such as cholesterol and glucose adsorption capacities.

The described process presented several advantages, including its short time, simplicity, and cost-effectiveness, using water as a solvent and requiring no further purification processing. Given this, the proposed approach conducted on a laboratory scale, with interesting results and processes in line with the concept of a circular bioeconomy in a sector of great global economic importance, has great potential from an industrial point of view; however, future research should focus on scaling up the process, monitoring all potential factors that could influence extraction efficiency.

## Figures and Tables

**Table 1:** Bioactive compounds and antioxidant activity of spent coffee grounds (SCGs), the liquid aqueous extract (LAE), and modified spent coffee grounds (MSCGs).

**Compound**	**SCGs**	**LAE**	**MSCGs**
CA (mg/100 g)	519.0 ± 0.3 ^a^	400.1 ± 0.3 ^b^	403.0 ± 1.6 ^b^
TPC (mg GAE/100 g)	1380.0 ± 0.1 ^a^	800.4 ± 0.1 ^b^	821.0 ± 0.1 ^b^
MEs (mg/100 g)	6640.8 ± 0.1 ^a^	2100.2 ± 0.1 ^c^	3439.6 ± 0.1 ^b^
**Antioxidant activity**	**SCGs**	**LAE**	**MSCGs**
DPPH (mg TE/100 g)	2015.2 ± 33 ^a^	154.5 ± 0.5 ^b^	93.2 ± 3.0 ^b^
ABTS (mg TE/100 g)	267.4 ± 2.2 ^b^	490.5 ± 0.1 ^a^	29.7 ± 0.7 ^c^

Data are the means of triplicate determinations ± standard deviation. Different superscript letters in the same line indicate significant differences (*p* ≤ 0.05) between means (Tukey’s test). CA, caffeine; TPC, total polyphenol content; MEs, melanoidins; ABTS, ABTS free radical scavenging antioxidant activity; DPPH, DPPH free radical scavenging antioxidant activity.

**Table 2:** Chemical characterization of spent coffee grounds (SCGs) and modified spent coffee grounds (MSCGs).

Compound (g/100 g)	SCGs	MSCGs
Moisture	4.4 ± 0.1 ^a^	2.9 ± 0.1 ^b^
Ash	1.9 ± 0.1 ^a^	0.9 ± 0.1 ^b^
Lipids	10.8 ± 0.1 ^a^	7.3 ± 0.2 ^b^
Proteins	1.2 ± 0.1 ^b^	2.9 ± 0.3 ^a^
Total fibers	65.5 ± 0.7 ^b^	73.0 ± 0.5 ^a^
Soluble fibers	1.4 ± 0.2 ^a^	1.6 ± 0.3 ^a^
Insoluble fibers	64.1 ± 0.9 ^b^	71.4 ± 0.4 ^a^
Cellulose	14.1 ± 0.6 ^b^	17.0 ±0.7 ^a^
Hemicellulose	23.0 ± 1.0 ^b^	27.4 ± 0.5 ^a^
Lignin	36.0 ± 1.2 ^b^	39.1 ± 1.4 ^a^

Data are the means of triplicate determinations ± standard deviation. Different superscript letters in the same line indicate significant differences (*p* ≤ 0.05) between means (*t*-test).

**Table 3:** Techno-functional properties of spent coffee grounds (SCGs) and modified spent coffee grounds (MSCGs).

Properties	SCGs	MSCGs
WHC (g/g)	3.5 ± 0.1 ^b^	3.7 ± 0.1 ^a^
OHC (g/g)	2.8 ± 0.1 ^a^	2.8 ± 0.1 ^a^
SC (mL/g)	3.9 ± 0.2 ^b^	4.2 ± 0.2 ^a^
HI (%)	11.0 ± 0.2 ^a^	10.6 ± 0.5 ^a^
EA (%)	2.0 ± 0.1 ^b^	3.3 ± 1.2 ^a^

Data are the means of triplicate determinations ± standard deviation. Different superscript letters in the same line indicate significant differences (*p* ≤ 0.05) between means (*t*-test). WHC, water holding capacity; OHC, oil holding capacity; SC, swelling capacity; HI, hygroscopicity; EA, emulsifying activity.

**Table 4:** Cholesterol and glucose adsorption capacity of spent coffee grounds (SCGs) and modified spent coffee grounds (MSCGs).

Properties	SCGs	MSCGs
CAC pH 2 (mg/g)	2.7 ± 0.9 ^a^	1.6 ± 0.3 ^b^
CAC pH 7 (mg/g)	1.6 ± 0.8 ^b^	8.0 ± 0.5 ^a^
GAC 50 mmol/L (mmol/L)	4.7 ± 0.8 ^b^	9.3 ± 1.1 ^a^
GAC100 mmol/L (mmol/L)	6.7 ± 3.2 ^b^	17.7 ± 2.6 ^a^

Data are the means of triplicate determinations ± standard deviation. Different superscript letters in the same line indicate significant differences (*p* ≤ 0.05) between means (*t*-test). CAC, cholesterol adsorption capacity; GAC, glucose adsorption capacity.

## Data Availability

The raw data supporting the conclusions of this article will be made available by the authors on request.
